# Biodistribution of Mesoporous Carbon Nanoparticles via Technetium-99m Radiolabelling after Oral Administration to Mice

**DOI:** 10.3390/nano11123260

**Published:** 2021-11-30

**Authors:** Maria Mamai, Dimitra Giasafaki, Evangelia-Alexandra Salvanou, Georgia Charalambopoulou, Theodore Steriotis, Penelope Bouziotis

**Affiliations:** 1 Institute of Nuclear & Radiological Sciences and Technology, Energy & Safety, National Centre for Scientific Research “Demokritos”, 15341 Athens, Greece; mariamamai94@gmail.com (M.M.); salvanou@rrp.demokritos.gr (E.-A.S.); 2 Institute of Nanoscience & Nanotechnology, National Centre for Scientific Research “Demokritos”, 15341 Athens, Greece; d.giasafaki@inn.demokritos.gr (D.G.); t.steriotis@inn.demokritos.gr (T.S.)

**Keywords:** CMK-1, mesoporous carbon nanoparticles, radiolabelling, Technetium-99m, oral administration, biodegradation, biodistribution

## Abstract

The use of ordered mesoporous matrices, and in particular carbon-based mesoporous nanoparticles has shown great potential towards enhancing the bioavailability of orally administered drugs. Nevertheless, elucidation of the in vivo absorption, distribution, and excretion of such carriers is essential for understanding their behaviour, and radiolabelling provides a very useful way to track their occurrence inside the body. In this work, uniform spherical CMK-1-type ordered mesoporous carbon nanoparticles have been radiolabelled with Technetium-99m (^99m^Tc) and traced after oral administration to mice. Ex vivo biodistribution studies showed that the radiolabelled nanoparticles accumulated almost exclusively in the gastrointestinal tract; complete elimination of the radiotracer was observed within 24 h after administration, with practically no uptake into other main organs. These findings along with the results from in vitro stability studies indicate that the spherical carbon nanoparticles examined could be safely used as drug carriers with minimal side effects, but also support the great value of radiolabelling methods for monitoring the particles’ behaviour in vivo.

## 1. Introduction

Oral drug delivery [1] constitutes the most widely used, cost-effective, non-invasive administration strategy for treating patients with comfort and simplicity [2]. However, the majority of commonly used medicines demonstrate a defective oral absorption profile, resulting from either their low aqueous solubility (that is essential for oral bioavailability [3]), or their poor stability in the harsh gastric environment [4]. Such limitations highlight the need for moving towards more efficient oral dosage forms for poorly water-soluble compounds [5,6,7]. At the same time, there has been a steadily growing interest in new multifunctional nanoparticles that could incorporate active agents for both therapeutic and diagnostic applications. A great variety of such nanomaterials has been explored so far (e.g., silicas and bioactive glasses, metals and metal oxides, polymers and lipids, quantum dots and carbon-based nanoparticles) [8,9]. In particular, mesoporous materials (pore widths between 2–50 nm) with tailorable pore properties have emerged as one of the most promising and advantageous biomolecule hosts that could also bear different functionalities useful not only for drug delivery, but also for targeting purposes [10]. Most of these mesoporous drug carriers, apart from the advantages of nano-confinement (drug amorphisation, stabilisation, and protection), have additional useful properties including (a) tunable texture (suitable pore size, surface area, pore volume) that allows significantly higher drug loadings [11], (b) chemical inertness, and (c) ease of functionalisation that may facilitate the encapsulation of several bioactive agents [12], but also impart specific responsive function upon endogenous and external stimuli (e.g., pH, redox, temperature changes and light) [13,14,15]. The most widely studied porous nanomatrices are silica-based materials [16,17,18,19], which however possess some important disadvantages (e.g., hydrothermal instability [20], potential toxicity [21] etc.), raising concerns about their safe and effective use as drug nanocarriers [22].

Mesoporous carbon nanoparticles (MCNs) [23], on the other hand, offer a non-toxic (as shown by several in vitro studies [14,24,25,26]) and structurally robust alternative with high thermal and mechanical stability [27]. Nevertheless, the potential of MCNs as drug delivery systems has not been fully explored yet, and in contrast to the massive work on mesoporous silica nanoparticles (MSNs) [28] they have only recently started to gain attention as components of therapeutic formulations [29]. Indeed over the past few years, a number of reports [30] have confirmed that mesoporous carbon materials can effectively encapsulate various poorly water-soluble drugs (e.g., ibuprofen [11,31,32], indomethacin [24], fenofibrate [33], simvastatin [34], carvedilol [35,36], itraconazole [37] etc.) but also enhance their dissolution and bioavailability. In addition to their great efficiency in terms of drug loading and controlled release, some MCNs also exhibit significant optoelectronic, photothermal-conversion and photoacoustic-generation properties, due to sp^2^ hybridization, while their surface can carry stimuli-responsive, fluorescent or targeting agents also through π-π stacking [38,39,40,41,42,43,44,45]. All these advantages make MCNs a promising, adjustable nanomedicine platform for real-time imaging, targeting and therapy.

Absorption of orally administered drugs is by itself a complex procedure [7] and a strong effort has been devoted to elucidating the interactions between drug formulations and the gastrointestinal track (GIT) [46]. The use of nano-vehicles complicates the process even further. A first step towards understanding the respective pharmacokinetics and a critical point for the development of effective drug delivery systems is monitoring the transport and biodistribution of the carriers in animal models [47,48,49]; however, biodistribution studies for MCNs are still lacking. Relevant investigations are so far limited to (0D) quantum dots [50,51,52] or to carbon nanostructures such as (1D) carbon nanotubes [53,54] and (2D) graphene [55]; 3D carbon particles (e.g., hollow core–mesoporous shell nanocapsules and CMK-type nanospheres) have been seldom studied, although they have shown high loading capacities and controlled release capabilities [11,14,27,32].

Radiolabelling represents an ideal tool for investigating the biodistribution of drug vehicles after their oral administration, while in vitro studies in the presence of simulated gastric and intestinal fluids, can provide information on both the degradation of the carriers and the release of the radiolabel [48,49]. A broad variety of radionuclides with half-lives ranging from a few minutes to several days are at the disposal of the radiochemist wishing to investigate the initial biodistribution and pharmacokinetics of a novel nanoconstruct/drug carrier. One of the radionuclides of choice for short-term assessment of the biokinetics of nanomaterials is the gamma-emitter Technetium-99m (^99m^Tc), due to its low energy gamma-photon emission (140 keV), suitable half-life (6 h), and availability from ^99^Mo/^99m^Tc generators [56,57,58]. ^99m^Tc is suitable for direct (chelator-free) radiolabelling of many nanoparticles (NPs), among which MCNs, as it bonds to surface groups at a neutral pH [59,60,61,62]. Padmanabhan et al. were the first to present a study of mouse gastrointestinal (GI) transit time, by using technetium-labelled activated charcoal diethylenetriainepentaacetic acid ([^99m^Tc]Tc-Ch-DTPA), which demonstrated that the total GI transit time is about 6 h in mice [47].

The present work aspires to provide significant insights into the transit, in vivo distribution, and excretion of spherical MCNs of CMK-1-type, after their oral administration. This is based on the effective radiolabelling of the MCNs, using radionuclides the decay of which can address several time-scales, as well as the assessment of the MCNs biodistribution at different time-points after their oral administration to healthy mice. To the best of our knowledge, this is the first study providing information on the in vivo kinetics and fate of MCNs with radiolabelling techniques using ^99m^Tc [63]. The obtained results combined with the in vitro stability studies are of high importance for the validation of the respective drug delivery systems, thus helping showcase their suitability for real therapeutic applications.

## 2. Materials and Methods

### 2.1. Materials

Warning! The ^99m^Tc isotope emits gamma radiation and presents serious health threats, thus it requires special radioprotective precautions during handling to reduce the risk of harm. Part of this research was conducted in a licensed radiochemical facility, which has all the necessary infrastructure and expertise to safely conduct experiments with radionuclides.

All reagents and solvents were purchased from Sigma-Aldrich (St. Louis, MO, USA) and used as received in the analytical grade. For preparation of the silica hard template (required for developing the ordered mesoporous carbon by nanocasting) cetyltrimethylammonium bromide (CTAB), triblock copolymer EO_106_PO_70_EO_106_ (Pluronic F127) and tetraethyl orthosilicate (TEOS 98%) were used, while sucrose (≥99.5%) was utilized as the carbon source. Technetium-99m, as Na[^99m^Tc]TcO_4_, was eluted from a commercial ^99^Mo/^99m^Tc generator (Mallinckrodt Medical B.V.). Radioactivity of the Na[^99m^Tc]TcO_4_ eluent and of all the radiolabelled species, was measured using a dose calibrator (Capintec, Ramsey, NJ). Thin-layer chromatography (TLC) silica gel 60 sheets (5 cm × 10 cm) were purchased from Merck (Darmstadt, Germany) and along with a Radio-TLC Scanner (Scan-Ram, LabLogic, Sheffield, UK) were used for the determination of radiolabelling yield/purity. Water was deionized to 18 MΩ·cm using an EASYpure^®^ water purification system (Barnstead International, Dubuque, Iowa). A gamma scintillation counter (Packard Cobra II, Canberra, Packard, Downers Grove, IL, USA), was used to measure the radioactivity of each organ and blood samples in the ex vivo biodistribution studies.

#### Synthesis of Ordered Mesoporous CMK-1 Carbon Spheres

The cubic periodic MCM-48 silica spheres were prepared via a modified Stöber method [64] by employing two different surfactants (cationic CTAB as the pore forming agent and nonionic Pluronic F127 as the grain size modulator) in a mixture of ethanol–aqueous ammonia solution and TEOS, as described elsewhere [14,16,32,65].

CMK-1 carbon spheres were obtained after double infiltration of the calcined MCM-48 template with acidic sucrose solution, followed by a two-step thermopolymerisation procedure, carbonisation at elevated temperature under inert atmosphere and finally dissolution of the silica “mould” with HF [14,32,65].

### 2.2. Characterisation

The morphology of the CMK-1 carbon spheres was investigated through Scanning Electron Microscopy (SEM) using a JEOL, JSM 7401F Field Emission (JEOL Ltd., Tokyo, Japan) Microscope equipped with a Gentle Beam mode. Dynamic Light Scattering (DLS) measurements were performed using an AXIOS-150/EX (Triton Hellas, Thessaloniki, Greece) (Triton Hellas) apparatus with a 30 mW laser source, and an Avalanche photodiode detector at a 90° angle, after dispersing the samples in aqueous medium. The pore structure periodicity of the mesoporous carbon was studied using Small Angle X-ray Scattering (SAXS) in transmission mode on a Rigaku, SmartLab X-ray diffraction system equipped with SAXS optics (λ = 1.54 Å). The scans were obtained from 0.06 to 8 degrees, with a speed of 20 s/step and a step of 0.02 degrees. The pore properties of the CMK-1 particles were evaluated by N_2_ adsorption–desorption measurements at 77 K performed on an Autosorb-1-MP, Quantachrome volumetric gas adsorption analyser. Prior to analysis, approximately 30 mg of the sample were appropriately outgassed for 24 h under high vacuum (10^−6^ mbar). The Brunauer–Emmett–Teller (BET) area value was calculated following the BET consistency criteria (ISO 9277:2010). The micropore volume was assumed to be the QSDFT (Quenched Solid Density Functional Theory) derived cumulative volume for pores smaller than 2 nm. The total (micro- and meso-) pore volume (TPV) was estimated at *p*/*p*_0_ = 0.90 (for pores with diameters <~20 nm), whereas the pore size distribution was deduced by using the N_2_-carbon QSDFT kernel for slit–cylindrical pores on the adsorption branch of the isotherm.

### 2.3. Radiolabeling of Carbon Nanoparticles

The radiolabelling procedure of CMK-1 was performed with ^99m^Tc via a direct method using SnCl_2_ as the reducing agent. Briefly, 200 μL of a phosphate buffer saline (PBS, pH = 10) was added in an Eppendorf and then 20 μL of CMK-1 (8 mg/mL dispersed in water) were also added to it. After the addition of 50 μL of a SnCl_2_ solution (8 mg in 250 μL HCl 30%, diluted to 5 mL after the addition of Millipore H_2_O) the pH of the mixture reached 2. The pH was thereafter adjusted to 6.5 by the addition of 300 μL PBS pH 10. Freshly-eluted Na[^99m^Tc]TcO_4_ was added (100 μL, ~1 mCi) and the reaction mixture was incubated at 60 °C for 60 min. The total radioactivity of the prepared sample was measured using a dose calibrator, while quality control of the radiolabelled CMK-1 (designated as [^99m^Tc]Tc-CMK-1) was performed with ascending ITLC-SG analysis using two different mobile phases, acetone and sodium citrate 0.1M, pH 5. A drop of the reaction solution (~5 μL) was applied at 1 cm from the bottom of a strip of ITLC-SG paper (1 cm × 12 cm) and allowed to dry. The strip was then placed in a beaker, which contained the mobile phase and was allowed to develop to 10 cm from the point of application. The strip was then removed from the beaker, allowed to dry, and was scanned on a Radio-TLC detector. Using acetone as the mobile phase, free [^99m^Tc]TcO_4_^-^ migrates to the front, while the radiolabelled CMK-1 and the potentially formed hydrolysed ^99m^Tc in the form of colloids ([^99m^Tc]TcO_2_) remain at the origin of the chromatogram. When developing the chromatogram in sodium citrate, [^99m^Tc]Tc-CMK-1 and free [^99m^Tc]TcO_4_^-^ are expected to move with the solvent front (Rf = 0.8–1.0), whereas [^99m^Tc]TcO_2_ remained at the origin (Rf = 0.0–0.2) [56,59,66]. By combining the results derived from the two developing systems (estimation of %[^99m^Tc]TcO_4_^-^ and %[^99m^Tc]TcO_2_), radiochemical purity (RCP) of [^99m^Tc]Tc-CMK-1 was calculated according to the following formula:

%RCP [^99m^Tc]Tc-CMK-1 = 100 − (%[^99m^Tc]TcO_4_^−^ + %[^99m^Tc]TcO_2_)

### 2.4. In Vitro Stability Studies

Stability of [^99m^Tc]Tc-CMK-1 was assessed in the presence of Simulated Gastric Fluid (SGF) and Simulated Intestinal Fluid (SIF) at 37 °C and Phosphate Buffered Saline (PBS, pH = 7.4) at room temperature. SGF was made by adding 3 g of NaCl in 1450 mL deionised H_2_0, while adjusting the pH to 1.2 with diluted HCl. For SIF (0.05 M, pH = 6.8), 0.34 g of Potassium Phosphate monobasic were diluted in 50 mL Millipore H_2_0 and the pH was adjusted with diluted NaOH. PBS (0.01 M, pH = 7.4) was prepared from PBS Tablets (Fisher BioReagents) diluted in Millipore H_2_0. For all stability tests, a sample of 10 μL of [^99m^Tc]Tc-CMK-1 was incubated with 40 μL of either SGF, SIF or PBS. Aliquots were taken from the mixtures at 1, 3 and 24 h and analysed by ITLC-SG analysis, as described above. All experiments were performed in triplicate, from three independent radiolabelling procedures. In order to investigate the non-labelled particle stability in the acidic gastric fluid, 50 mg of pristine CMK-1 were immersed into 200 mL of SGF and incubated under stirring at 37 °C for 24 h. The sample (CMK-1_SGF) was thoroughly washed and dried at 40 °C and characterized with SEM, SAXS and N_2_ sorption measurements.

### 2.5. Biodistribution Studies

Animals used for the biodistribution studies were obtained from the breeding facilities of the Institute of Biosciences and Applications, NCSR “Demokritos”. Our experimental animal facility is registered according to the Greek Presidential Decree 56/2013 (Reg. Number: EL 25 BIO 022) in accordance with the European Directive 2010/63, which is harmonized with national legislation, on the protection of animals used for scientific purposes. All applicable national guidelines for the care and use of animals were followed. The study protocol was approved by the Department of Agriculture and Veterinary Service of the Prefecture of Athens (Protocol Number: 1607/11-04-2018). The animals were housed in air-conditioned rooms in an IVC unit (Tecniplast S.p.A., Buguggiate, Italy), under a 12 h light/dark cycle and allowed free access to food and water.

The in vivo behaviour of the radiolabelled CMK-1 was studied in normal Carworth Farms White (CFW) Swiss Webster mice (*n* = 3 mice per time-point, average animal weight 35 g). [^99m^Tc]Tc-CMK-1 was administered orally via a plastic gavage microapparatus (200 μL, ~70 μCi). For the ex vivo biodistribution experiment, the animals were euthanized at 1, 3, 6 and 24 h post-administration, and the organs and tissues of interest (blood, liver, heart, kidneys, stomach, intestines, spleen, muscle, lung, bone, pancreas, brain) were removed, weighed, and measured in an automatic gamma counter. The radioactivity remaining in the tail, as well as the background counts, were subtracted, while the radioactivity decay was auto corrected by the counter. A standard dose of the injected solution was used in all calculations. The uptake of the radiolabelled carbon NPs in each organ/tissue was expressed as the mean percentage of injected dose per gram of tissue ± standard deviation (%ID/gr ± SD).

## 3. Results and Discussion

### 3.1. Morphological Properties

**Figure 1:**
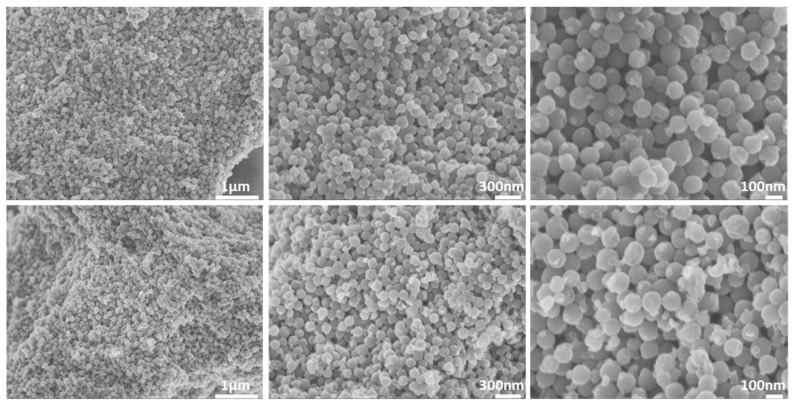
SEM images of the CMK-1 carbon spheres before (**top**) and after incubation with SGF (**bottom**).

As revealed from the SEM images in Figure 1 (top), the pristine CMK-1 sample comprises uniform spherical mono-dispersed nanoparticles, with an average size of 100–120 nm. No morphological variation was observed after treatment with the acidic SGF (pH = 1.2), as shown in Figure 1 (bottom). DLS (intensity weighted) measurements revealed a mean hydrodynamic diameter of ~240 nm for both pristine and SGF treated samples.

### 3.2. Structural Properties

**Figure 2:**
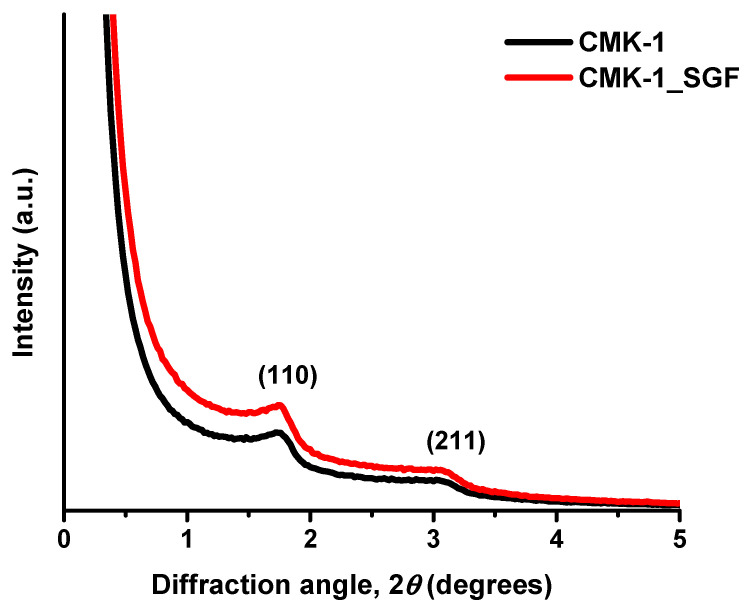
SAXS pattern of the CMK-1 carbon spheres before and after incubation with SGF.

The SAXS diffractogram of the as-produced carbon particles exhibited the typical structure of the CMK-1-type ordered mesoporous carbon, thus confirming its successful synthesis. More specifically, as presented in Figure 2, the pattern of CMK-1 particles, shows two clear peaks attributed to the (110) and (211) reflections of the 3D tetragonal structure with *I*4_1_/*a* symmetry [65,67,68,69]. As also shown in Figure 2, there was no practical difference in the structure of the pristine particles (black line) after incubation with the acidic SGF (red line).

### 3.3. Pore Properties

**Figure 3:**
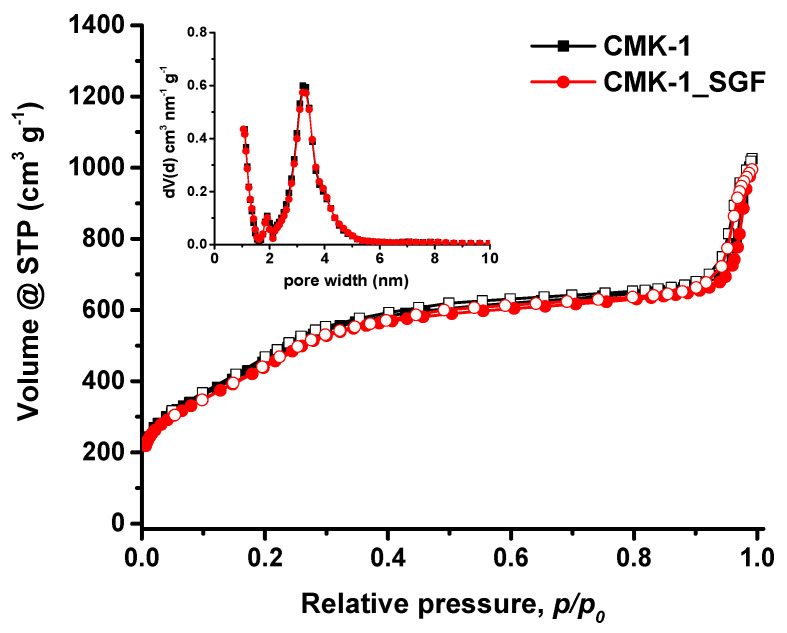
N_2_ adsorption (full symbols)-desorption (open symbols) isotherm and pore size distribution (PSD) (inset) of the CMK-1 carbon spheres before and after incubation with SGF.

The pore properties of CMK-1 and CMK-1_SGF particles were assessed by N_2_ adsorption–desorption measurements at 77 K. Figure 3 shows the obtained isotherms along with the corresponding pore size distributions (inset). In accordance with SEM and SAXS results, both samples showed practically identical results, confirming the stability of the carbon particles in SGF. The isotherms are of type IVb, based on the International Union of Pure and Applied Chemistry (IUPAC) classification [70,71], i.e., typical of ordered mesoporous materials with pore sizes smaller than ~4 nm. In addition to the stepwise behaviour with no hysteresis loop (indicative of uniform narrow mesopores), the increased nitrogen adsorption at low relative pressures (*p/p_0_* < 0.01) also suggests the presence of a significant amount of microporosity. A large external surface area and a considerable secondary pore volume (i.e., >0.5 cm^3^/g for pores with diameters between 20–160 nm) are also evident at high relative pressures due to the carbon spheres’ packing.

The pore properties of the CMK-1 and CMK-1_SGF samples are summarised in Table 1. It can be seen that they exhibit a high BET area of about 1500 m^2^/g, a large total pore volume of 1.0 cm^3^/g (at *p/p_0_* = 0.90) for pores smaller than 20 nm (diameter) and uniform mesopores with a mean size of approximately 3.2 nm, (Figure 3, inset). The total pore volume (at *p/p_0_* = 0.99) including meso- to macro- pore volume (20–160 nm) was estimated to be around 1.55 cm^3^/g, thus indicating considerable additional volume due to the interparticle voids formed by the agglomerated carbon spheres.

### 3.4. Radiolabeling of CMK-1 and In Vitro Stability Assessment

In order to develop an efficient drug delivery system, the respective pharmacokinetic and biodistribution profiles need to be thoroughly investigated. The radiolabelling of CMK-1 nanoparticles with ^99m^Tc provided us with the means to assess their in vivo behaviour at pre-determined time-points after their oral administration to healthy CFW mice. To our knowledge, this is the first time that mesoporous carbon NPs were radiolabelled and investigated as described above. Quantification of radiolabelled nanoparticles relies on the fact that the tracer is always associated with the NPs. In our study, we need to investigate whether or not the radionuclide remains bound to the NPs during its transit through the GI tract. Then, we can accurately reach a conclusion with regard to the in vivo kinetics of the CMK-1 nanoparticles.

Technetium-99m is eluted from a ^99^Mo/^99m^Tc generator as a pertechnetate anion ([^99m^Tc]TcO_4_^-^), the chemical reactivity of which is negligible, thus synthesis of ^99m^Tc-radiolabeled compounds requires its reduction to lower oxidation states, with reducing agents such as stannous chloride. Excess amounts of stannous chloride lead to undesirable radiocolloid formation and subsequent accumulation in the organs of the reticuloendothelial system (RES) due to macrophage uptake. On the other hand, concentration of stannous chloride below the optimum level for reducing pertechnetate leads to its incomplete reduction from the heptavalent state. After meticulous investigation of radiolabelling conditions, we have achieved excellent radiolabelling yields (>95%) with negligible colloid formation, confirming that stannous chloride concentration used was appropriate.

**Figure 4:**
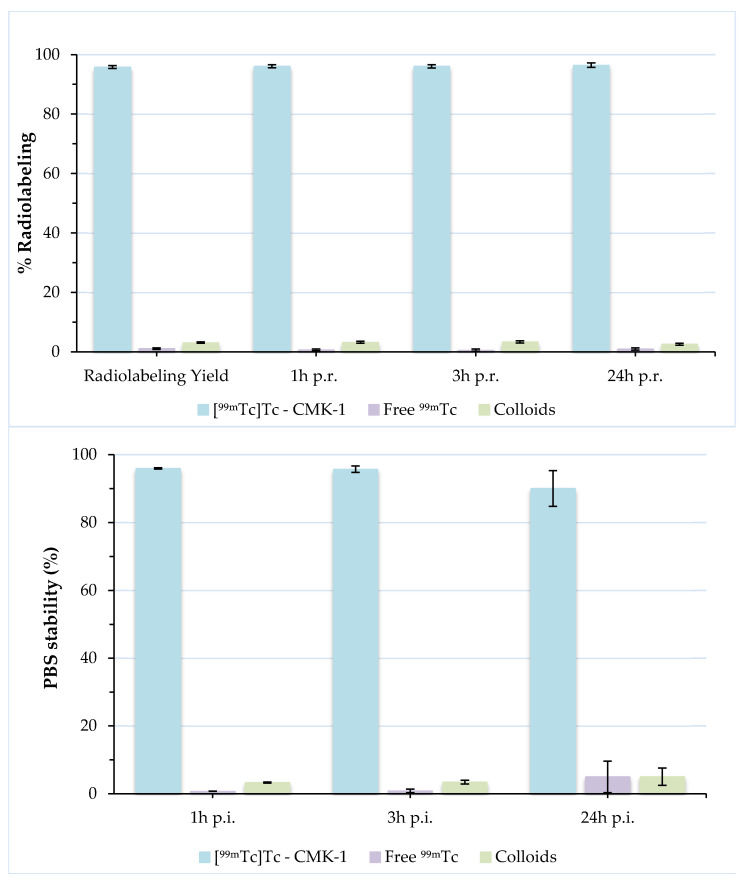
Radiochemical stability of [^99m^Tc]Tc-CMK-1 in the presence of PBS pH 7.4 up to 24 h post-incubation (p.i.).

Direct radiolabelling of CMK-1 with ^99m^Tc relies on the interaction between deprotonated hydroxyl groups present on the carbon surface and the Tc(V) ions [56,72,73]. Stable labelling is thus achieved without the use of chelators, which may be detached from CMK-1 in vivo. Various labelling conditions were tested, with respect to time and temperature. The highest radiolabelling yields were achieved after 60 min incubation at 60 °C (pH ~7). The radiochemical purity was evaluated by ITLC-SG and was found to be ~96%. Further assessment of [^99m^Tc]Tc-CMK-1 at 3 h and 24 h post-preparation showed that there was only a slight release of the radioisotope (<2% free ^99m^Tc at 24 h). Nanoparticles radiolabelled with ^99m^Tc via the direct labelling approach have demonstrated high stability, as shown by the low degree of transchelation of the isotope in the presence of PBS [59,74,75]. The in vitro stability of [^99m^Tc]Tc-CMK-1 was assessed by incubation in PBS for 1, 3 and 24 h, at RT, and showed that the ^99m^Tc-MCNs were stably labelled, exhibiting a low degree of ^99m^Tc release up to 24 h post-incubation (~90% intact [^99m^Tc]Tc-CMK-1) (Figure 4).

**Figure 5:**
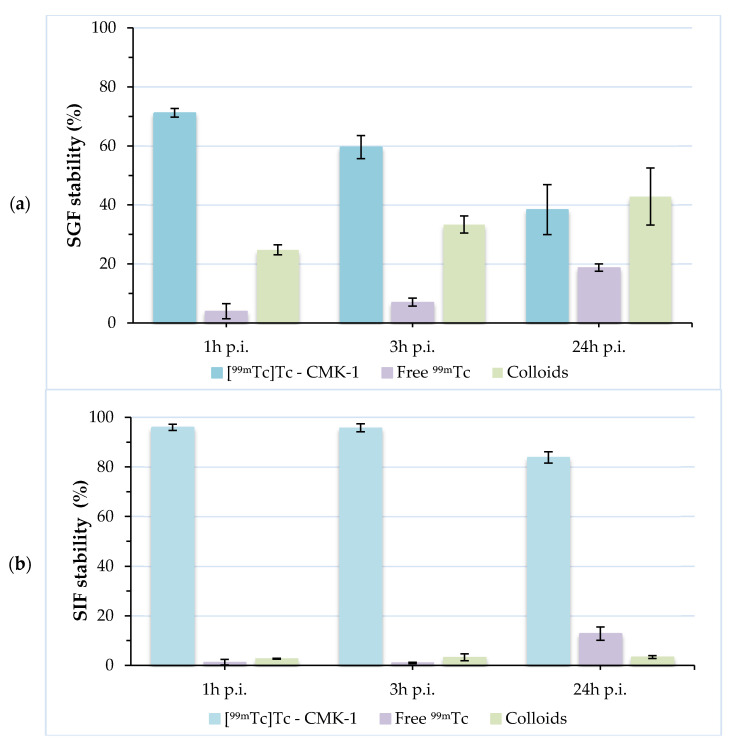
Radiochemical stability of [^99m^Tc]Tc-CMK-1 in the presence of: (**a**) simulated gastric fluid and (**b**) simulated intestinal fluid at 1, 3 and 24 h post-incubation (p.i.).

When evaluating an orally administered formulation, its stability must be assessed in the presence of gastric and intestinal fluids. These stability studies are mandatory, as they demonstrate the degree of attachment of the radiolabel to the nanoconstruct. Incubation of [^99m^Tc]Tc-CMK-1 in SGF (pH 1.2) showed a release of ~30% of the radiolabel in the forms of pertechnetate and [^99m^Tc]TcO_2_·nH_2_O at 1 h post-incubation. This may be due to the fact that a certain number of surface functional (hydroxyl) donor groups are in protonated form, leading to partial desorption of the ^99m^Tc species and hydrolysis to insoluble [^99m^Tc]TcO_2_ [76]. Further release of the radiolabel (~40% intact [^99m^Tc]Tc-CMK-1) was observed at 24 h post-incubation. On the contrary, [^99m^Tc]Tc-CMK-1 is quite stable up to 24 h post-incubation in the presence of SIF (~84% intact [^99m^Tc]Tc-CMK-1 at 24 h post-incubation) (Figure 5). The observed radiolabel release in SGF could not be attributed to in vivo degradation of the CMK NPs, as these were found to be unaltered in acidic media (Figure 1, Figure 2 and Figure 3). In the presence of SGF, radiolabelling integrity is affected and ^99m^Tc is released from the nanostructure. The radioactivity uptake in the stomach can thus be attributed to both the radiolabelled CMK-1 as well as the pertechnetate and [^99m^Tc]TcO_2_ species formed at low pH.

### 3.5. Biodistribution Studies

**Figure 6:**
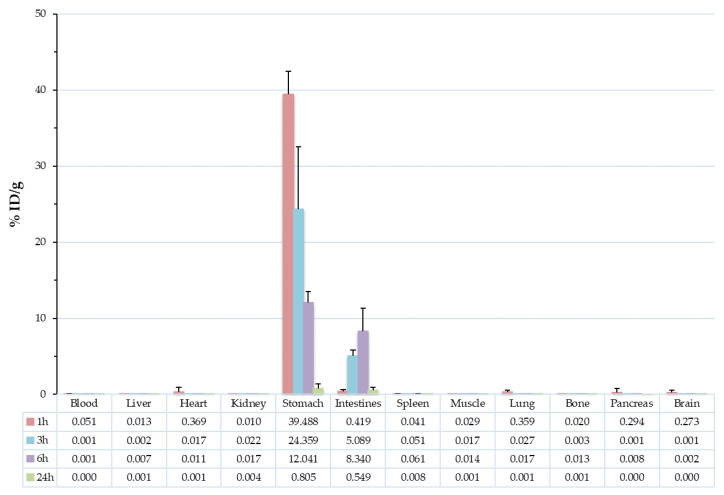
Biodistribution of [^99m^Tc]Tc-CMK-1 in CFW mice at 1, 3, 6 and 24 h post-administration.

Ex vivo biodistribution studies for the determination of the in vivo characteristics of the radiolabelled CMK-1 were carried out in healthy female CFW mice, after their oral administration. After oral administration of [^99m^Tc]Tc-CMK-1, the mice were euthanized by isoflurane inhalation at 1, 3, 6 and 24 h post-administration and their blood, major organs and tissues were studied simultaneously (in order to refer to the same degree of radioactive decay); in all cases no sign of toxicity was observed. As shown in Figure 6 and Table 2, [^99m^Tc]Tc-CMK-1 was almost exclusively distributed in the stomach and intestines. At 1 h post-administration, the radiotracer was mainly found in the stomach (39.49 ± 2.97% ID/g), while intestinal uptake slowly increased in time (0.42 ± 0.24% ID/g vs. 8.34 ± 3.01% ID/g at 1 and 6 h post-administration, respectively). By 24 h post-administration, complete elimination of the radiotracer was observed. Throughout the duration of the biodistribution study, practically no uptake was observed in the other major organs, thus we expect any possible side effects from drug administration via loading of the developed MCNs to be minimal. Similar results were shown by other groups investigating the in vivo kinetics of drug nanocarriers with radiolabelling or fluorescent-labelling techniques [47,77]. As mentioned previously, in the presence of SGF radiolabelling, integrity is affected and ^99m^Tc is released from the nanostructure, but not distributed in vivo (no radioactivity has been detected in other organs or tissues). The radioactivity uptake in the stomach and consequently intestines can thus be attributed to the radiolabelled CMK-1, as well as the pertechnetate and colloids formed at low pH.

## 4. Conclusions

Investigation of the biodistribution of MCNs is a necessary step for their use as effective drug delivery carriers. The in vivo tracking of MCNs has been investigated by radiolabelling with ^99m^Tc, which proved to be a simple and straightforward procedure. The radiolabelled [^99m^Tc]Tc-CMK-1 was monitored by ex vivo biodistribution studies up to 24 h post-oral administration, which determined its almost exclusive presence in the gastrointestinal tract, with complete absence from all other organs investigated for radiotracer uptake. At 24 h post-administration, [^99m^Tc]Tc-CMK-1 was completely washed out of the organism, with <1% ID/g remaining in the GI tract. In the case of stomach uptake, the radioactive label corresponds to the intact radiolabelled CMK-1, as well as to free pertechnetate and [^99m^Tc]TcO_2_, which are formed at the low pH of the stomach environment (as shown by stability studies in SGF). On the other hand, almost all of the radioactivity present in the intestines can be attributed to the intact [^99m^Tc]Tc-CMK-1. It should be noted that CMK-1 particles do not degrade in the stomach as the SGF incubation experiments revealed. In this respect, although part of the surface-attached ^99m^Tc was released in the stomach, burst drug release caused by nanoparticle dissolution is highly improbable. This study has shed light on the biodistribution of MCNs, and thus their suitability as an orally administered drug delivery vehicle, proving also the important role of radiolabelling techniques in the elucidation of the in vivo behaviour of such nanostructures.

## Figures and Tables

**Table 1:** Pore properties of the CMK-1 carbon spheres before and after incubation with SGF (pH = 1.2).

Sample	S_BET_ (m^2^/g)	TPV (cm^3^/g) at *p/p_0_ =* 0.90	V_micro_ (cm^3^/g)	V_meso_ (cm^3^/g)	Pore Width (nm)
**CMK-1**	1513	1.03	0.22	0.81	3.20
**CMK-1_SGF**	1506	1.01	0.21	0.80	3.20

S_BET_: BET area, TPV: total pore volume (pores < 20nm), V_micro_: micropore volume, V_meso_: mesopore volume (=TPV − V_micro_) and Pore Width: mean pore diameter obtained by QSDFT analysis.

**Table 2:** Biodistribution data of [^99m^Tc]Tc-CMK-1 in CFW mice at 1, 3, 6 and 24 h post-administration.

	60 min		3 h		6 h		24 h	
	%ID/g	STDV	%ID/g	STDV	%ID/g	STDV	%ID/g	STDV
Blood	0.05	0.04	0.00	0.00	0.00	0.00	0.00	0.00
Liver	0.01	0.01	0.00	0.00	0.01	0.01	0.00	0.00
Heart	0.37	0.58	0.02	0.00	0.01	0.01	0.00	0.00
Kidney	0.01	0.00	0.02	0.01	0.02	0.01	0.00	0.00
Stomach	39.49	2.97	24.36	8.17	12.04	1.49	0.81	0.60
Intestines	0.42	0.24	5.09	0.74	8.34	3.01	0.55	0.42
Spleen	0.04	0.06	0.05	0.01	0.06	0.01	0.01	0.01
Muscle	0.03	0.02	0.02	0.00	0.01	0.00	0.00	0.00
Lung	0.36	0.20	0.03	0.04	0.02	0.02	0.00	0.00
Bone	0.02	0.02	0.00	0.00	0.01	0.02	0.00	0.00
Pancreas	0.29	0.47	0.00	0.00	0.01	0.00	0.00	0.00
Brain	0.27	0.29	0.00	0.00	0.00	0.00	0.00	0.00

## Data Availability

Data is contained within the article.
